# Anomalies échocardiographiques chez l'hémodialysé chronique: prévalence et facteurs de risqué

**DOI:** 10.11604/pamj.2014.18.216.4438

**Published:** 2014-07-15

**Authors:** Mariam Ezziani, Adil Najdi, Souad Mikou, Anis Elhassani, Mohammed Amine Akrichi, Hakim Hanin, Mohammed Arrayhani, Tarik Sqalli Houssaini

**Affiliations:** 1 Service de Néphrologie, CHU Hassan II, Fès, Maroc; 2 Laboratoire d’épidémiologie, de recherche clinique et de santé communautaire, Faculté de Médecine et de Pharmacie de Fès, Maroc

**Keywords:** Hémodialyse chronique, hypertension artérielle, échocardiographie transthoracique, hypertrophie ventriculaire gauche, chronic hemodialysis, hypertension, transthoracic echocardiography, left ventricular hypertrophy

## Abstract

**Introduction:**

Les complications cardio-vasculaires sont la principale cause de mortalité et de morbidité chez les patients hémodialysés chroniques. Le but de cette étude est de décrire les différentes atteintes cardiaques à l’échocardiographie transthoracique et de dégager les facteurs associés aux principales atteintes.

**Méthodes:**

Etude transversale monocentrique incluant des patients adultes hémodialysés depuis plus d'un an et ayant bénéficié d'une échocardiographie transthoracique durant l'année 2012. Notre étude descriptive puis analytique a porté sur l'analyse des données démographiques, cliniques, biologiques et échocardiographiques.

**Résultats:**

50 patients ont été colligés, 32 femmes et 18 hommes. Leur âge moyen est de 55 ± 14 ans avec une ancienneté moyenne en hémodialyse de 93 ± 55 mois. Les anomalies échocardiographiques retrouvées sont dominées par l'hypertrophie ventriculaire gauche chez 56% des patients et les valvulopathies chez 80%. Les calcifications valvulaires ont été notées dans 14% des cas, une dilatation du ventricule gauche dans 12%, une hypertension artérielle pulmonaire dans 10% et une péricardite dans 6% des cas. Une seule dysfonction du ventricule gauche a été notée et la fraction d’éjection systolique moyenne est de 68,9 ± 9,5%. L'analyse statistique de variance des différentes anomalies montre que l'hypertrophie ventriculaire gauche est positivement corrélée à l'hypertension artérielle (p = 0,046) et que les pressions artérielles moyennes systoliques (p = 0,005) et diastoliques (p = 0,035) sont significativement plus élevées chez les patients avec une hypertrophie du ventricule gauche.

**Conclusion:**

Les anomalies échocardiographiques sont fréquentes chez les hémodialysés chroniques notamment l'hypertrophie ventriculaire gauche qui est fortement liée à la présence d'une hypertension artérielle.

## Introduction

La mortalité annuelle des maladies cardiovasculaires chez les patients en dialyse est substantiellement plus élevée que dans la population générale. Elles sont responsables d'environ 50% des décès et de 30% des hospitalisations des patients en dialyse. L'anomalie morphologique la plus fréquente est l'hypertrophie ventriculaire gauche (HVG). Cette dernière est retrouvée chez 60 à 75% des patients qui arrivent au stade terminal de l'insuffisance rénale chronique et chez 60 à 90% de ceux régulièrement dialysés [1, 2].

L'HVG est un processus de remodelage adaptatif bénéfique puisqu'elle permet au ventricule gauche de maintenir une tension pariétale stable face aux surcharges de pression secondaire à l'hypertension artérielle (HTA) et de volume secondaire à l'hypervolémie [3, 4]. L’échocardiographie transthoracique (ETT), examen non invasif, disponible, actuellement largement utilisé dans l’évaluation de la structure et de la fonction cardiaque, demeure un outil de référence dans le bilan des atteintes cardiaques chez les patients traités par hémodialyse périodique afin de définir ceux à haut risque cardiovasculaire [5].

La fréquence de l'atteinte cardiaque chez l'hémodialysé chronique dépend de plusieurs facteurs de risque qui peuvent être intriqués, à savoir l'hypertension artérielle, l'anémie, l'inflammation chronique, l'hyperparathyroïdie, l'homocystéinémie et bien d'autres [6]. Par ailleurs, un traitement intensif des facteurs de risque de l'HVG permet une nette régression de la masse du ventricule gauche et réduit donc toutes les causes de mortalité cardiovasculaire [7]. Le but de notre travail est de déterminer la prévalence des différentes anomalies cardiaques objectivées à l'ETT chez une population d'hémodialysés chroniques et de dégager les facteurs de risque associés aux principales atteintes.

## Méthodes

Il s'agit d'une étude transversale monocentrique incluant des patients adultes hémodialysés depuis plus d'un an ayant bénéficié d'une ETT durant l'année 2012. Pour chacun de nos patients, nous avons analysé les données démographiques, cliniques, biologiques et échocardiographiques. Les données cliniques ont été recueillies à partir des dossiers des malades et comportent: l’âge au moment de l’étude, le sexe, la durée en hémodialyse exprimée en mois, la néphropathie initiale, les pressions artérielles systolique (PAS) et diastolique (PAD), les caractéristiques de l'abord vasculaire, la prise de poids inter dialytique (PPID) et la dose de dialyse mesurée par le Kt/V.

Les paramètres biologiques étudiés étaient: la calcémie, la phosphorémie, les taux de cholestérol, de HDL-cholestérol, de LDL-cholestérol et de triglycérides, le taux d'hémoglobine, la ferritinémie, la c-réactive protéine (CRP) et l'albuminémie. Pour chacun de ces paramètres, nous avons calculé la moyenne annuelle. Le taux de parathormone intacte (PTHi) retenu est celui effectué au moment de l’étude. L'ETT a été effectuée entre deux séances d'hémodialyse lorsque la volémie était la plus voisine de la normale afin d’éviter le facteur de surcharge hydro-sodée. Elle a permis l’étude des structures cardiaques (myocarde, péricarde et endocarde), la recherche des différents types d'anomalies morphologiques (calcifications, dilatations et hypertrophie) et le retentissement fonctionnel systolique et/ou diastolique. Les paramètres cardiaques étudiés étaient les suivants: le septum inter-ventriculaire (SIV), la paroi postérieure (PP), la fraction d’éjection (FE) pour étudier la fonction systolique, le diamètre télédiastolique du ventricule gauche (DTD) et la masse du ventricule gauche (MVG) et le diamètre de la veine cave inférieure.

L'analyse statistique a été réalisée par le logiciel SPSS 17.0 par le laboratoire d’épidémiologie et de recherche clinique de la faculté de médecine et de pharmacie de Fès. Les variables quantitatives ont été exprimées en moyenne ± écart-type et les variables qualitatives en pourcentage. Les tests t de Student et le chi 2 ont été utilisés pour la comparaison des variables quantitatives et qualitatives respectivement. Une valeur p < 0,05 est considérée significative.

## Résultats

Nous avons colligé 50 patients hémodialysés chroniques dont 32 femmes et 18 hommes. Leur âge moyen est de 55 ± 14 ans avec une ancienneté moyenne en hémodialyse de 93 ± 55 mois. Cinq patients sont hypertendus et l'anémie est retrouvée dans 48% des cas. Les caractéristiques démographiques, cliniques et biologiques de notre population sont représentées dans leTableau 1 et le Tableau 2.

**Tableau 1:** Les caractéristiques démographiques et cliniques des patients

Paramètres	
Age moyen (ans)	55 ± 14
Sexe ratio (F/H)	32/18
Durée en hémodialyse (mois)	93 ± 55
**Néphropathie initiale : (%)**	
Diabète	10
HTA	22
Néphrite tubulo-interstitielle chronique	20
Glomérulonéphrite chronique	6
Indéterminée	42
**Abord vasculaire (Fistule artério-veineuse native)**	
Distal	80%
Proximal	20%
Prise de poids interdialytique (PPID) (Kg)	2,6 ± 0,7
PAS moyenne (cmHg)	11,63 ± 2,01
PAD moyenne (cmHg)	7,01 ± 1,19
Kt/V hebdomadaire	3,7 ± 0,9

**Tableau 2:** Les caractéristiques biologiques des patients

Paramètres biologiques	Moyenne ± écart-type
Calcémie (mg/l)	94 ± 6
Phosphorémie (mg/l)	38 ± 13
Parathormone (pg/ml)	789 ± 551
Albuminémie (g/l)	37 ± 3,4
Cholestérolémie (g/l)	1,66 ± 0,4
LDL-cholestérol (g/l)	1 ± 0,31
HDL-cholestérol (g/l)	0,35 ± 0,12
Triglycérides (g/l)	1,5 ± 0,64
Hémoglobinémie (g/dl)	11 ± 1,8
Ferritinémie (mg/l)	432 ± 201
C -réactive protéine (mg/l)	7,9 ± 5,6

**Figure 1:**
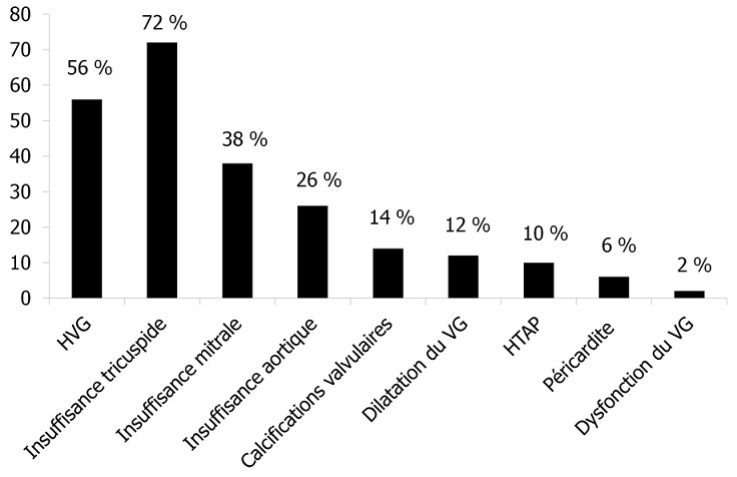
Les différentes anomalies cardiaques à l’échocardiographie

Les anomalies échocardiographiques retrouvées sont dominées par l'hypertrophie ventriculaire gauche chez 56% des patients et les valvulopathies chez 80% des patients. Les autres atteintes sont décrites dans la Figure 1. En effet, l'analyse descriptive de ces différentes anomalies a noté une prédominance de l'HVG. Cette dernière est définie par une augmentation des parois ventriculaires (SIV et PP). Ainsi nous retrouvons une valeur moyenne du SIV de 12,16 ± 3,04 mm et de la PP de 10,57 ± 2,5 mm.

Les valvulopathies (incluant les maladies mitrales, aortiques et tricuspidiennes) sont objectivées dans 80% des cas. Cependant, les calcifications valvulaires mitro-aortiques ont été notées dans 14% des cas seulement. Un seul cas de dysfonction systolique a été noté avec une fraction d’éjection systolique à 40%, alors que la dilatation du VG est retrouvée dans 12% des cas. L'hypertension artérielle pulmonaire (HTAP) est retrouvée chez 10% des cas et la péricardite chez 6% des cas.

L'analyse statistique de variance des différentes anomalies cardiaques montre que l'HVG est positivement corrélée à la présence d'une HTA (p = 0,046) et que les pressions artérielles moyennes systolique (p = 0,005) et diastolique (p = 0,035) sont significativement plus élevées chez les patients avec HVG.. Cependant, les autres facteurs étudiés notamment: l’âge, l'ancienneté en hémodialyse, les perturbations du bilan phosphocalcique, l'hyperparathyroïdie, l'hypo albuminémie, les dyslipidémies, l'anémie et la CRP ne favorisent pas le développement de l'HVG dans notre étude (Tableau 3).

**Tableau 3:** Les facteurs de risque associés à l'HVG

*Variables*	*Groupe HVG +(n = 28)*	*Groupe HVG* –(n = 22)	*P*
Age (ans)	54 ± 16	56 ± 12	NS
Durée en hémodialyse (mois)	93 ± 61	93 ± 47	NS
PPID (Kg)	2,69 ±0,78	2,50 ± 0,61	NS
IMC (Kg/m2)	22,2 ± 4,5	22,6 ± 3,0	NS
Kt/V hebdomadaire	3,6 ± 1,0	3,8 ± 0,7	NS
HTA actuelle	17,86%	0%	0,03
PAS (cmHg)	12,3 ± 2,1	10,8 ± 1,6	0,005
PAD (cmHg)	7,3 ± 1,1	6,6 ± 1,3	0,035
Calcémie (mg/l)	94 ± 6	93 ± 6	NS
Phosphorémie (mg/l)	36±14	40 ± 11	NS
Parathormone (pg/ml)	864 ± 624	701 ± 447	NS
Vitamine D3 (ng/ml)	50 ± 47	42 ± 31	NS
Albumine (g/l)	39 ± 3	36 ± 3	NS
Cholesterol (g/l)	1,7 ± 0,6	1,7 ± 0,4	NS
Triglycerides (g/l)	1,5 ± 0,5	1,5 ± 0,7	NS
LDL (g/l)	1 ± 0,3	0,9 ± 0,3	NS
HDL (g/l)	0,32 ± 0,1	0,38 ± 0,1	NS
Anémie	46,4%	72,7	NS
Hémoglobinémie (g/dl)	10,7±1,6	10,5±1,8	NS
Ferritinémie (mg/l)	441±201	422±205	NS

## Discussion

Les affections cardiaques dans leur ensemble sont la cause la plus fréquente de morbidité et de mortalité chez les patients insuffisants rénaux chroniques traités par dialyse [8]. Chez le dialysé le risque de mortalité cardiovasculaire a été comparé à celui de la population générale. Il est 500 fois plus élevé chez les patients âgés de 25 à 35 ans et de cinq fois chez les patients âgés de plus de 85 ans [2]. L’échocardiographie transthoracique est un outil non invasif, disponible et reproductible qui permet un diagnostic précis des anomalies cardiaques.

Les principales anomalies morphologiques des cardiomyopathies retrouvées dans la littérature sont l'HVG et la dilatation du VG [4]. Ces dernières peuvent s'accompagner d'une altération de la fonction systolique ou diastolique avec comme conséquence clinique une insuffisance cardiaque, un trouble du rythme, voire la mort subite. Initialement, l'HVG est un processus de remodelage adaptatif aux surcharges de volume et de pression et bénéfique puisqu'elle permet de maintenir un niveau de stress pariétal stable. Cependant, elle peut devenir mal adaptée et délétère à cause de la mort cellulaire (apoptose) secondaire à une surcharge ventriculaire continue, à une réduction de la densité capillaire et à la fibrose myocardique [7]. En effet, 28% des patients insuffisants rénaux chroniques développent une HVG au début de la dialyse, ceci en rapport avec l'abord vasculaire (fistule artério-veineuse), la surcharge hydro-sodée et l'anémie [9, 10]. Dans la littérature, l'HVG est retrouvée dans 75% dans la série de London [11] et dans 73,9% dans la série de Foley [12] alors que dans notre série elle est de 56%. La fréquence des affections cardiaques chez les hémodialysés chroniques peut être expliquée par plusieurs facteurs de risque propres à cette population (HTA, fistule artério-veineuse, anémie, rétention hydro-sodée). Elle peut être aussi liée à des facteurs de risque communs à l'ensemble de la population générale que sont l’âge, le tabac, le diabète et les dyslipidémies.

Ainsi, dans notre série, comme dans plusieurs études [13], la présence d'une hypertension artérielle systolique ou diastolique a été notée comme facteur significatif favorisant l'HVG chez l'hémodialysé chronique. L'anémie entraine une augmentation du débit cardiaque suite à une élévation de la fréquence cardiaque et du volume d’éjection systolique. Ce qui crée des conditions de surcharge volémique chronique responsable d'une dilatation du VG et d'un épaississement septal à l’échocardiographie [14]. Cette influence a été mise en évidence par plusieurs auteurs, notamment London et Coll [15] qui retrouvent ainsi une relation inverse entre la concentration de l'hémoglobine, d'une part, la dilatation et la masse ventriculaire gauche, d'autre part. Dans notre série, l'anémie ne s'est pas révélée associée au développement de l'HVG.

L'inflammation chronique a été décrite, dans la littérature, comme facteur de risque de l'atteinte cardiaque aussi bien chez l'hémodialysé chronique que dans la population générale [8]. Par ailleurs, l'hypo albuminémie qui s'accompagne d'une augmentation de la mortalité est ajoutée à la liste des marqueurs du risque cardiovasculaire. Dans notre étude, le taux de CRP et le taux d'albumine ne semblent pas être corrélés à cette atteinte.

## Conclusion

Les complications cardiaques sont fréquentes dans la population des hémodialysés, elles sont dominées par l'hypertrophie ventriculaire gauche avec comme facteur de risque majeur la présence de l'hypertension artérielle.
